# YAP/Hippo Pathway and Cancer Immunity: It Takes Two to Tango

**DOI:** 10.3390/biomedicines9121949

**Published:** 2021-12-20

**Authors:** Dimitrios Matthaios, Maria Tolia, Davide Mauri, Konstantinos Kamposioras, Michalis Karamouzis

**Affiliations:** 1 Department of Medical Oncology, Rhodes General Hospital, 85100 Rhodes, Greece; 2 Department of Radiotherapy, School of Medicine, University of Crete, 71500 Heraklion, Greece; mariatolia1@gmail.com; 3 Department of Medical Oncology, University of Ιoannina, 45110 Ιoannina, Greece; dvd.mauri@gmail.com; 4 Department of Medical Oncology, The Christie NHS Foundation Trust, Manchester M20 4BX, UK; kambkons1@yahoo.gr; 5 Molecular Oncology Unit, Department of Biological Chemistry, Medical School, National and Kapodistrian University of Athens, 11527 Athens, Greece; m.karamouzis@imibe.org

**Keywords:** Hippo pathway, YAP, LATS1/2, TAZ, immunity, cancer, immunotherapy

## Abstract

Hippo pathway with its main molecule YAP is a crucial pathway for development, tissue homeostasis, wound healing, tissue regeneration, and cancer. In this review, we discuss the multiple effects of the YAP/Hippo pathway in the immune system and cancer. We analyzed a series of effects: extracellular vesicles enhanced immunity through inhibition of LATS1/2, ways of modulation of the tumor microenvironment, YAP- and TAZ-mediated upregulation of PDL1, high expression of YAP and PDL1 in EGFR-TKI-resistant cells, enhanced YAP activity in inflammation, and the effect of the Hippo pathway on T cells, B cells, Tregs, macrophages, and myeloid-derived suppressor cells (MDSCs). These pleiotropic effects render the YAP and Hippo pathway a key pathway for exploitation in the future, in order to enhance our immunotherapy treatment strategies in oncology.

## 1. Introduction

Cancer immunotherapy has dramatically changed the clinical landscape of the treatment in many malignancies. Non-small cell lung cancer, melanoma, and renal cancer are examples where immunotherapy either alone or in combination with chemotherapy substantially improved overall survival.

However, even with these encouraging improvements, acquired resistance and disease progression is an issue, which needs to be solved.

Moreover, given the dogma that more effective treatment should be given early in the disease progress, there is an unmet need to give more effective therapy in the first-line setting.

Antibodies targeting PDL1 (Programmed death-ligand 1) and CTLA4 (Cytotoxic T-Lymphocyte Associated Protein 4) are important checkpoint inhibitors enhancing T cell killing of tumor cells, making them efficacious treatments in many solid tumors, such as lung cancer, melanoma, renal carcinoma, and others, while some types of solid tumors are resistant to immunotherapy.

The Hippo pathway, originally discovered through a series of genetic mosaic screens for genes augmenting cell proliferation and organ size in Drosophila, is a critical pathway in mammalian cells [1]. Canonical signaling through the Hippo pathway core components (MST1/2, LATS1/2, YAP, and TAZ) is significant for development and tissue homeostasis while defective signaling through the Hippo pathway has been implicated in multiple pathologies, including cancer. Hippo pathway “key players”, such as YAP (yes-associated protein) and TAZ (transcriptional coactivator with PDZ-binding motif), are key drivers of wound healing, tissue regeneration, and tumor progression.

There is accumulating evidence that YAP/TAZ expression may confer resistance to immunotherapy by upregulating PDL1 expression [2] and by regulating MDSC cells (myeloid-derived suppressor cells) [3,4,5,6,7].

In this review, we aim to analyze the role of the YAP/Hippo pathway affecting immunity in solid tumors, by elucidating its effect on critical “players” of the immune context both in the tumor and the tumor microenvironment. Addressing and dissecting the key impact points of the Hippo pathway and YAP in the immune system will provide future challenges and perspectives in exploiting Hippo pathway blocking, in order to provide synergistic therapies with current immunotherapies by potentiating efficacy and overcoming resistance.

## 2. Ev-Mediated Enhanced Immunity through Inhibition of Yap Inhibitory Kinases Lats1-2

Extracellular vesicles (EVs) are structures regulating the interaction and communication between cells [8]. They are released by cells, including tumor cells, and may contain lipids, nucleic acids, and proteins [9]. EVs have been isolated from many cell types, including cancer cells from cell lines [10,11,12]. Tumor-derived EVs are known to influence tumor angiogenesis [13], the recruitment of cancer-associated fibroblasts [14], can regulate metastasis, and may play potential roles in immune evasion [15]. Moreover, tumor EVs can be used as carrier vesicles to transfer parent cell material to recipient cells. There is also evidence that EVs derived from cancer cells can transfer activated EGFR to endothelial cells [12].

**Figure 1:**
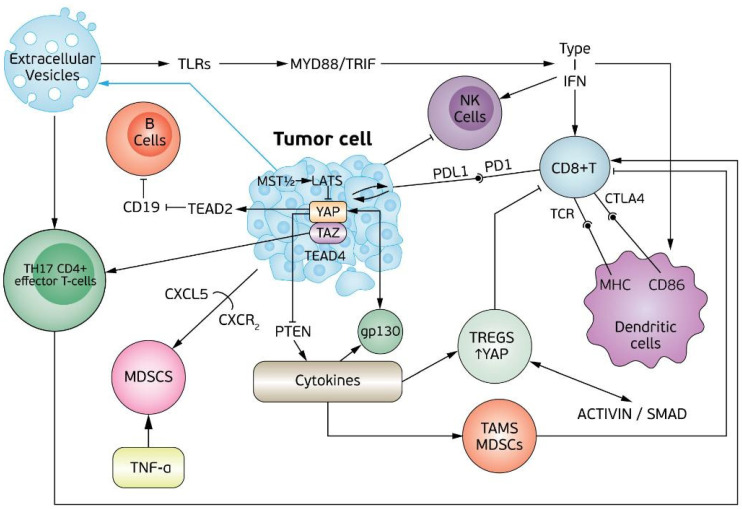
Hippo pathway/YAP in tumor cell and interactions with key molecules of immunity: B cells, CD8+ T cells, Th17 CD4+ effector T cells, MDSCs (myeloid-derived suppressor cells), TAMs (tumor-associated macrophages), TREGs (regulatory T cells), MHC (major histocompatibility complex), NK cells (natural killer cells), extracellular vesicles, TLRs (toll-like receptors), type I IFN (type I interferons).

Moroishi et al. provided a novel mechanism of a Hippo pathway-mediated effect on immunogenicity. They showed that by deleting LATS1 and LATS2 (large tumor suppressor 1 and 2), tumor immunogenicity was modulated by enhancing the content of nucleic acid released in extracellular vesicles (EVs) by tumor cells. Host immune cells recognize these nucleic acids by toll-like receptors and invoke type I interferon (IFN) receptors, which in turn affect NK (Natural Killer) cells, dendritic cells, and T cells. Especially, dendritic cells, when activated, induce CD8 T cells [16] (Figure 1).

In order to fully interpret these conflicting results, we should keep in mind that there may be species-specific differences in YAP/TAZ transcriptional targets. Helena J. Janse van Rensburg et al. found that there is a difference between human and murine cell lines since there are gene targets that are differentially regulated by TAZ, including PDL1 [2]. This emphasizes the need to compare the impact of Hippo signaling in the immune system across species along with the ultimate goal of focusing on human in vivo studies.

## 3. Modulation of the Tumor Microenvironment

Yap signaling can have an impact on the tumor microenvironment by upregulating cytokines. There is evidence from Wang et al. that CXCL5 which is upregulated through YAP, can attract MDCSs (myeloid-derived suppressor cells) to the tumor by heterotypic CXCL5 binding to CXCR2 receptors [17]. Murakami et al.’s findings are in line with the aforementioned, using a mouse model of pancreatic ductal adenocarcinoma (PDAC) along with human samples of PDAC [18]. In the same context, in an ovarian mouse model, YAP upregulates TNFa, inhibits cytotoxic T cells, and recruits MDSCs [3]. There is also growing evidence in the literature that enhancement of the M2 phenotype of tumor-associated macrophages could lead to tumor microenvironment modification [19,20] (Figure 1).

## 4. Upregulation of PDL1 Expression

YAP and TAZ directly upregulate the expression of the immune checkpoint molecule programmed death ligand-1 (PD-L1), and as a consequence suppress the T cell function [2]. Taha et al. showed that the TAZ/YAP/TEAD4 complex enhances PDL1 promoter activity [21]. Lee et al. knocked down YAP in lung adenocarcinoma cells, which in turn reduced PDL1 levels [22]. The authors found that the EGFR-TKI-resistant PC9 cells had significantly lower levels of YAP and PDL1 compared to the parental PC9 adenocarcinoma cells. They proceeded to silence YAP, which in turn lowered the expression of PDL1 in the resistant PC9 cells. Lee et al. revealed that YAP regulates the transcriptional level of PDL1, and that the TEAD/YAP complex binds to PDL1 promoter. They also found that knockdown of PDL1 decreased wound healing and cell proliferation in resistant EGFR cells. Overall, these data favor an independent oncogenic function of PDL1. It seems that YAP in the context of the Hippo pathway has a major role in controlling the expression of PDL1 in lung cancer, providing a link to EGFR resistance and PDL1 and rendering it an attractive target to overcome EGFR-TKI resistance in lung adenocarcinoma [22]. Tung et al. also reported that there is a kind of loop in which PDL1 overexpression increases YAP transcriptional activity and levels along with YAP-induced TKI drug resistance in NSCLC [23]. Helena J. Janse van Rensburg et al. tried to study the impact of YAP and TAZ in the regulation of immune-related genes. They found that PDL1 is a target of the Hippo pathway. Upstream kinases of the Hippo pathway, such as LATS1/2 and mammalian STE20-like kinase 1 and 2 (MST1/2), downregulate PDL1 levels in contrast to the upregulation of PDL1 levels through YAP and TAZ activity. TAZ/YAP/TEAD complex seems to increase the activity of the PDL1 promoter. The authors also point out that PDL1 upregulation caused by TAZ results in inhibition of T cell activity. What is also of great interest is the fact that PDL1 and TAZ feedback is not the same across multiple cell lines, which is possibly explained due to differences between mouse and human PDL1 promoters. Helena J. Janse van Rensburg et al. performed a second screen using murine cell lines, concluding that a diversity of TAZ targets may exist through the species, highlighting the role of the Hippo pathway in modifying immune system activity, and establishing a role of this pathway in human cancer immune evasion [2].

In melanoma, Kim and colleagues showed that YAP inhibition led to regulation of PDL1 expression and directly inhibited cytotoxic T cells, which improved BRAF inhibition efficacy and as result patient survival [24].

Hsu et al. described a correlation between PDL1 levels and YAP in human pleural mesothelioma (MPM) since they are co-expressed in immunohistochemistry. The authors concluded that inhibition of YAP downregulates PDL1 expression in MPM [25] (Figure 1).

## 5. Inflammation and Cancer

There is evidence that enhanced YAP activity in tissues with inflammation may contribute to tumorigenesis. APC-mutated colon cancer, which is the main genetic deficit of familial adenomatous polyposis coli (FAP) syndrome, has high expression of gp130 and is more sensitive to IL-11, IL-6, and siL-6R [26]. The sustained high gp130 levels drive YAP upregulation, which in turn upregulates gp130 through TEAD4, forming an autoregulatory feedback loop [26]

Kim et al. established a murine genetic hepatocellular carcinoma (HCC) model by deleting Mst1 and Mst2 in hepatocytes and through a series of experiments they examined the effects on Mcp1 (a marker of inflammation) and YAP. Deletion of Mst1 and Mst2 in hepatocytes (DKO) led to HCC development, highly upregulated Mcp1 expression, and massive infiltration of macrophages with mixed M1 and M2 phenotypes. Macrophages or deletion of Mcp1 in DKO mice markedly reduced hepatic inflammation and HCC development. The authors also found that Yap knocked down and nullified induction of Mcp1 expression and restored normal liver growth in the Mst1/Mst2 DKO mice. They also found that Mcp1 is a direct transcriptional target of YAP in hepatocytes and identified a strong gene expression correlation between Yap targets and Mcp1 in human HCCs. In conclusion, they identified the Hippo-Yap signaling pathway as a key upstream regulator of Mcp1, linking the hepatic growth control function of Hippo signaling with the regulation of inflammatory responses [27,28].

TH17 CD4+ effector T-cells (TH17 cells) are known to be important for inflammatory-related diseases including cancer [29]. Geng et al. showed the Hippo pathway to be important in TH17 and Treg (regulatory T cells) cell lineage determination. Knockout of Mst1/2 or overexpression of Taz in T cells increased the number of TH17 cells and decreased the number of Treg cells. Furthermore, mice with Taz-deficient T cells had more Treg cells and were resistant to induction of TH17-dependent inflammation [30]. Thus, Taz induction during T cell differentiation may be an important factor in tumor immune responses [31].

## 6. Yap and T Cells

Yasuda et al. showed that cytotoxic T lymphocytes with MST1 deficiency had lower levels of FOXO1 and FOXO3A, which negatively regulate CD8 T cells [19]. As a result, these MST1 knockout cytotoxic T cells (CTLs) showed increased cytotoxicity both in vitro and in vivo in mouse thymoma models. [32]. It is also important to note that high YAP expression in CD4 T lymphocytes induces differentiation into Treg cells [33]. There is growing evidence that TAZ expression in CD4 T lymphocytes decreases Tregs differentiation by, in parallel, enhancing Th17 differentiation [30]. Buglioni et al. highlighted the dual role of YAP/TAZ in TILs (tumor-infiltrating lymphocytes) and cancer cells. They found that although in TILs, TAZ/YAP levels correlated with an increased response to neoadjuvant chemotherapy due to increased clonal expansions of CD8+ T cells, this was not the case in cancer cells, where TAZ/YAP levels correlated with poor prognosis [34]. CD8+ T cells lacking Mst1 provide better protection against implanted tumors in animal models [35]. Interestingly, Mst1-deficient CD8+ T cells express elevated levels of T-bet, which is a transcription factor that was originally discovered as a lineage marker of TH1 cells because it can establish TH1 differentiation and inhibit polarization of other CD4+ T cell subsets, such as TH2 or TH17 cells, and its target effector molecule IFN-γ, suggesting that Mst1 may have a negative regulatory role in antitumor CD8+ T cell responses. The increased T-bet expression in Mst1-deficient CD8+ T cells was correlated with reduced levels of FoxO1 [32], consistent with the previously established role of FoxO1 in repressing T-bet expression [36]

There is evidence that Akt may promote whereas Mst kinases could dampen β-catenin stabilization in T cells. This is in contrast to FoxO signaling: Akt inhibits whereas Mst promotes nuclear translocation of FoxO proteins. Therefore, the relative strength of Akt and Mst kinase activities in different T cell subsets may highly influence the outcome of β-catenin and FoxO signaling [37].

Ni et al. examined the role of YAP in the regulation of Treg cells. They found that YAP is highly expressed in Tregs. Knocking down YAP and Tregs failed to suppress the activation of the immune system both in vitro and in vivo. Ni et al. found that the dimeric member of the TGFb cytokine superfamily known as activin signaling is increased by YAP through activation of the component of the activin receptor complex. YAP signaling seems to be present in Tregs and could also increase SMAD/TGFb signaling and promote Treg differentiation. The authors found that the blockage of the SMAD/YAP/activin axis substantially decreased the growth of tumors in mice, including a highly aggressive melanoma model. This experimental treatment also enhanced the antitumor efficacy of an antitumor vaccine, suggesting that the targeting of this YAP/activin/ SMAD axis can be used to improve anticancer immunotherapy efficacy, providing hope for future strategies that will combine anti-YAP treatments with other immunotherapy drugs. The same authors also found that YAP-deficient Tregs display reduced expression of several genes known to be important in the signaling pathway triggered by the anti-inflammatory cytokine TGFβ. Interestingly, one of the genes most downregulated in the absence of YAP was that encoding the signaling component of the activin receptor complex known as Acvr1c. These findings open new roads since it provides insight for future experiments that will examine the upstream blocking of Treg maturation by blocking Acvr1c. They also found that deletion of Yap1 in T cells somewhat enhances both Th1 and Th17 development but most impressively diminishes the generation of induced Tregs under conditions of limited TGFβ. YAP deficiency also negatively affects the suppressive function of Tregs. The inability of Tregs to suppress immunity in vivo in the absence of YAP was dramatically illustrated by B16 melanoma tumor model experiments. The poorly immunogenic tumor failed to grow in mice with Treg-specific Yap deletion, which displayed markedly enhanced indicators of proinflammatory antitumor immunity compared with WT controls. This improved deployment of antitumor immunity was seen together with a markedly lower Treg presence in the tumor microenvironment, findings also observed on Treg-specific YAP deficiency across other distinct tumor models [38] (Figure 1).

## 7. Yap and B Cells

The main role of B cells fighting tumor cells is to present antigens to T cells and directly kill them [39,40,41]. Bai and colleagues found that YAP suppresses B cell differentiation through activation of TEAD2. Upon TEAD2 activation, this event transcriptionally suppresses cd19 levels through binding to the 30UTR consensus motif. This binding results in the activation of BCR signaling, differentiation of peripheral B cells, and endocytosis [42].

## 8. YAP and Macrophages

Lee and colleagues found that YAP/TAZ regulate about 66 genes related to differentiation, immunity, cell development, and metabolism, such as myoblast determination protein (MyoD), lymphocyte function-associated antigen 1 (LF-A1), PPARg, and the finger of the cerebellum 1 (Zic1) and about 69 other genes regulating macrophages [43]. In hepatocellular carcinoma, YAP provokes the migration of macrophages both in vivo and in vitro [44]. Guo et al. studied the association between tumor-initiating cells (TICs) and M2 macrophages at the tumor initiation stage in hepatocellular carcinoma [4]. AKT and EGFR activate YAP in tumor cells and recruit TIC-associated macrophages (TICAMs) to liver TICS by enhancing Ccl2/Csf1 secretion in the initial stage and also converts hepatocytes to TICs [4]. YAP-induced TICAMs eradicate YAP+TICs and inhibit the clearance of TICs, and as a result affect tumorigenesis and the survival of TICs [4]. Huang et al. found that YAP induces M2 TAM polarization in colorectal cancer, which promotes their tumor-initiating ability [20] and correlates with poor prognosis in many cancers [45,46,47]. The same authors concluded that blocking YAP in combination with 5-fluorouracil reduced tumorigenesis and prevented TAM polarization and TAM-mediated resistance to the treatment [20]. Cui et al. found that Src-PI3K-YAP signaling is a mechanism of the angiogenesis caused by macrophage-associated immunosuppression [48]. There is growing evidence that downstream Src signaling, including the PI3K and MAPK pathways, promotes YAP upregulation [49]. Overall, analyzing all these data, we can conclude that Src represses Hippo kinases, leading to YAP activation, either with interaction with its upstream cell surface receptors or by affecting downstream signaling pathways.

## 9. YAP and MDSCs

Tumoral YAP expression is a predictor of poor prognosis in patients with colorectal cancer, owing to its correlation with the presence of MDSCs (myeloid-derived suppressor cells) and reduced survival of patients with colorectal cancer [50].

MDSCs are heterogenous immature myeloid cells with the ability to differentiate into macrophages, DCs (dendritic cells) and neutrophils and they promote immunologic tolerance. There is evidence that they inhibit CD8 cytotoxic T cell activity [51,52]. Wang et al. found that MDSCs were recruited to the tumor microenvironment (TME) in prostate carcinoma models and promoted tumorigenesis in a YAP-dependent manner [17]. The authors found that YAP activation, and especially its nuclear localization, induced the secretion of Cxcl5, which is a ligand for Cxcr2-expressing MDSCs, attracting other MDSCs by Cxcl5-Cxcr2 signaling as a result. MDSCs cells impeded the proliferation of the T cell population, which resulted in a tumor increase [17]. In a model of pancreatic ductal adenocarcinoma that was KRAS:p53 mutated, YAP increased CSF1-3 and IL6 levels, which drove MDSCs differentiation and accumulation. This has a negative effect on T cell activation and the reprogramming of macrophages along with poor survival of patients [3]. In colorectal cancer, a strong association between the density of CD33 MDSCs and YAP and phosphatase tensin homolog (PTEN) levels has been described [53]. There is evidence that the MDSCs population is expanded by the inhibition of PTEN through YAP expression. When PTEN is suppressed, this in turn results in the activation of pAKT, Pp65, and COX-2 signaling and in the promotion of cytokine granulocyte macrophage colony-stimulating factor production. This also affects the differentiation of MDSCs [53]. When the population of MDSCs is expanded, this results in the activation of T cells, which in turn results in a tumor increase [54,55]. In high-grade ovarian serous carcinoma (HGOSC), YAP was found to regulate an atypical protein kinase (aPKC) enzyme that contributes to cell proliferation and cancer development, the protein kinase C iota type, which in turn contributes to the immunosuppression of the tumor microenvironment [18,56]. PRKCI activation increased the upregulation of YAP and its nuclear localization and in turn increased the expression of TNFa [56], which led to MDSCs recruitment and impairment of cytotoxic T cell infiltration and NK activation [57,58] (Figure 1).

At the level of microRNAs that are known to regulate the expression of oncogenic pathways, Meng et al. studied the role of mir-21 in lung cancer mice cells along with its impact on YAP levels [59]. It is known that over-expression of miR-21-5p by mesenchymal stem cell-secreted extracellular vesicles promotes the development of lung cancer [60]. Bioinformatics analysis showed that RUNX1 transcription factor is one of the downstream targets of miR-2 [61]. There was also evidence that RUNX1 can inhibit YAP [62]. Meng et al. found that miR-21 maintained MDSCs accumulation in the tumor microenvironment and promoted the immunosuppressive ability of MDSCs in Lewis lung cancer-bearing mice by downregulating RUNX1and upregulating YAP, providing the rationale for future studies that will incorporate targeting of miR-21 in MDSCs [59].

## 10. YAP/TEAD Inhibitors

YAP/TEAD inhibitors can be classified into three categories: category 1 involves compounds that attack upstream YAP/TAZ activators, category 2 involves compounds that directly attack the YAP/TAZ-TEAD complex, and category 3 involves compounds that attack downstream YAP/TAZ targets (Table 1, [63]).

Category 2 consists of compounds that directly block YAP or TEAD. When TAZ and YAP are paired with TEAD transcription factors, upregulation of the expression of several oncoproteins is observed. When TEAD does not have a DNA binding domain, YAP is no longer oncogenic [64]. As a result, the prevention of YAP/TAZ–TEAD interaction or the inhibition of TEAD constitutes an effective and promising treatment strategy that needs to be further validated in clinical trials [65]. Verteporfin is a direct YAP inhibitor, and it also disrupts YAP–TEAD interaction. Apart from Verteporfin, several drugs with a benzisothiazole-dioxide scaffold, which binds to TEAD and disrupts the YAP/TAZ–TEAD interaction, have been studied and we await these drugs entering clinical trials, especially in malignant pleural mesothelioma and breast and lung cancers, where the Hippo pathway is disrupted [66].

Another potential molecule derived from the co-regulator VgII4 that disrupts the YAP–TEAD interaction has been studied in animal models [67,68,69]. In the same context, there is research that aims to find disruptors of the interaction between YAP/TAZ and TEAD. These disruptors target the palmitate-binding pocket (PBP). A potent inhibitor that targets the PBP is K-975 [70]. This molecule also disrupts YAP–TAZ–TEAD interaction with significant activity in malignant pleural mesothelioma. As a result, there is ongoing research aiming to construct chemical scaffolds targeting PBD and blocking TEAD and YAP–TAZ–TEAD interaction. These molecules, also called destabilizers, unfold the TEADs YAP/TAZ-binding domain. As an additional tool to this direction, ongoing research is trying to couple either TEAD blockers or PBP-occupying agents with a proteolysis-targeting chimera (PROTAC) [71] driving TEAD proteasomal degradation.

## 11. Clinical Implications and Future Directions

YAP/Hippo pathway is a key regulator of the immune system, playing a vital role in regulating immunity at multiple levels. A critical question is whether all this accumulating evidence can be clinically exploited. The majority of the compounds listed in Table 1 were tested in preclinical models. The other crucial issue is that the most tested compounds are indirect YAP inhibitors. This is something that has both advantages and disadvantages. The advantages are: (a) we have safety experience of these compounds, (b) these agents may have combinatorial effects with the treatment modalities already used in clinical practice (chemotherapy, immunotherapy, targeted therapies), while the disadvantage is that YAP targeting is indirect. In the group of Category 1, several molecules are under investigation, such as statins, which inhibit the nuclear translocation of YAP and target HMG-CoA reductase. An issue for consideration regarding statins is that they require high concentrations to inhibit YAP and they also have pleiotropic effects [72,73,74]. Of course, it is of concern that this type of inhibitors cannot fully recapitulate the effects of the category 2 direct compounds. On the other hand, direct YAP/TAZ/TEAD inhibitors are currently under investigation [75]. Verteporfin is a promising agent that directly blocks YAP activity. There is evidence in the literature that Verteporfin, by blocking YAP, can surpass the resistance due to TKI, RAFi, and other chemotherapy regimens [76,77,78,79,80,81].

However, Verteporfin only blocks YAP-TEAD binding at high micromolar concentrations and has YAP-independent effects; therefore, we need more data and await clinical trials to see if it is a clinically relevant YAP inhibitor.

Another field of research is focused on the post-translational modifications (PTMs) performed on the YAP/TAZ/TEAD transcriptional complexes. This should be investigated in the context of TEAD targeting, which can have identical and similar effects to YAP targeting [82,83,84,85,86]. The next step is to discover whether small molecule inhibitors that target PTMs could efficiently block the activities of YAP/YAZ/TEAD transcription complexes.

Regarding the focus of our review on the immune system, we analyzed the pleiomorphic effect of the YAP/Hippo pathway at multiple levels regulating the immune system. What we described is that there is an orchestrated multi-level effect of the YAP/Hippo pathway at various levels of the immune system. There is evidence of extracellular vesicle-mediated enhanced immunity through inhibition of Yap inhibitory kinases Lats1-2. Moreover, we discussed the evidence that Yap signaling can have an impact on the tumor microenvironment by upregulating cytokines. We also discussed the fact that YAP and TAZ directly upregulate the expression of the immune checkpoint molecule programmed death ligand-1 (PD-L1), and as a consequence suppress the T cell function. This is also a phenomenon observed in the EGFR-TKI-resistant setting and can be reversed through YAP inhibition. Furthermore, we discussed the role of inflammation in this context. The sustained and high gp130 levels drive YAP upregulation, which in turn upregulates gp130 through TEAD4, forming an autoregulatory feedback loop. We also know that regarding T cells, high YAP expression in CD4 T lymphocytes induces differentiation into Treg cells. YAP is highly expressed in Tregs. Knocking down YAP, Tregs, failed to suppress the activation of the immune system both in vitro and in vivo. Deletion of Yap1 in T cells somewhat enhances both Th1 and Th17 development but most impressively diminishes the generation of induced Tregs under conditions of limited TGFβ. YAP deficiency also negatively affects the suppressive function of Tregs. In the context of TAMs, YAP induces M2 TAM polarization, which promotes their tumor-initiating ability and correlates with poor prognosis in many cancers. We also analyzed how MDSCs are recruited to the tumor microenvironment (TME) and promote tumorigenesis in a YAP-dependent manner. MDSCs prevented T cell proliferation and promoted tumor progression.

The rationale of targeting the Hippo pathway is strongly supported by the aforementioned data of regulation of PDL1 expression along with immune-suppressive cytokines, driving immune evasion. However, we should keep in mind that there are reports that YAP/TAZ regulation of PDL1 is human specific [2]. This renders the modeling of the YAP/Hippo pathway with syngeneic or genetically engineered mouse models (GEMMs) similar but not exactly identical. Furthermore, although we know that YAP/TAZ is highly expressed across most solid tumors, this is not the case with blood malignancies [87,88].

It is also true that nowadays, our clinical practice is mostly biomarker driven in order to maximize our molecular targeted strategies. The expression or activation status of YAP/TAZ might be a predictor of YAP inhibitors’ efficacy, and such stratification might be important for anti-YAP/TAZ therapy. This is something future clinical trials should incorporate in their study design.

## 12. Conclusions

In conclusion, we analyzed the pleiotropic effect of the YAP/Hippo pathway in the immune system, trying to elucidate its possible role in tumorigenesis and immunotolerance through a plethora of evidence in the literature. Translational and clinical trials that will encompass YAP inhibitors and other molecules targeting the Hippo pathway, alone or in combination with current immunotherapy regimens in cancer treatment, are needed to answer the question of whether the YAP/Hippo pathway can help meet the unmet need of further improving the current good results of immunotherapy in oncology.

## Figures and Tables

**Table 1:** YAP/TEAD inhibitors.

YAP/TEAD Inhibitors Category	Compounds
Category 1 (compounds that attack YAP/TAZ upstream activators)	MEK inhibitors (*PD98059*, *U0126 and trametinib*), MAPK inhibitors (CAY10561 and FR180204), γ-secretase inhibitors (GSIs, DAPT or dibenzazepine), EGFR inhibitors (*erlotinib*, *AG-1478*),GPCR inhibitors, VEGFR inhibitors (*SU4312*, *Apatinib*, *Axitinib and pazopanib*), adenyl cyclase, γ-secretase complex inhibitors (*DAPT or dibenzazepine*), Agrin inhibitors, G-protein coupled receptors (GPCRs) inhibitors or stimulators (*losartan*, *dihydrexitine*, *gallein*), integrin blocking agents (*RGD peptide*, *cilengitide*, *function-blocking antibodies—BHA 2.1 and clone AIIB2*,*QLT0267*),forskolin, cyclic nucleotide phosphodiesterases (PDE) (*theophylline*, *IBMX*, *ibudilast and rolipram*), FAK inhibitors (*PF-562271 and PF-573228*, *CT 707*, *PF431396*), SFK inhibitors (*Dasatinib*, *PP2*, *SU6656*, *AZD0530*, *SKI-1*),FAK inhibitors (*PF-562271*, *PF-573228*, *CT-707*, *PF431396*), PI3K inhibitors (*Wortmannin/LY294002*, *BX795*), mTOR inhibitors (*temsirolimus*, *MLN0128*, *torin 1 inhibitor*), Mevalonate pathway inhibitors (statins, zoledronic acid and GGTI-298, actin modulators (*latrunculin A*, *cytochalasin D*), Myosin-myosin light-chain kinase inhibitors (*blebbistatin*, *ML-7*), Rho kinase inhibitors (*Y27632*, *toxin C3*), p21 activated kinase (PAK) family kinases (*IPA3*, *PF-03758309*), Phosphatase inhibitors (*okadaic acid or calyculin A*), SHP2 inhibitors, mitochondrial complex I inhibitors (*metformin*, *phenformin*), AMPK activators (*A769662*, *AICAR*), HDAC inhibitors (*panobinostat*, *quisinostat*, *dacinostat*, *vorinostat*, *Trichostatin A*, *CG200745*), HDAC inhibitor panobinostat with BET (bromodomain and extra-terminal) inhibitor I-BET151, BET inhibitors (JQ1),
Category 2 (Compounds directly targeting YAP/TAZ or TEAD family)	Verteporfin, TEAD stabilizers, TEAD destabilizers/degraders, YAP cyclic peptide (peptide 17), cystine-dense peptide (TB1G1), a peptide derived from the co-regulator Vgll4, fenamate drugs (palmitate, flufenamic acid), fenamate analogs, vinylsulfonamide derivatives, DC-TEADin02, K-975, quinolinols, PBP–occupying compounds are coupled to proteolysis targeting chimera (PROTAC),
Category 3 (Compounds against downstream YAP/TAZ targets)	A37 targeting ALDH1A1,aminooxyacetate (AOA) targeting GOT1, celecoxib targeting COX2, NS398 targeting COX-2, TP-0903 conferring Axl inhibition, WZ400 inhibiting NUAK2, CXCL5 neutralizing antibody, blocking CXCL5 receptor with the inhibitor SB255002, Jagged-1 neutralizing antibody, cyclopeptide RA-V (deoxybouvardin) reduces integrin ligands CTGF and CYR61,BCL-xL inhibition using navitoclax, thiostrepton that reduces FOXM1 levels, TG2 inhibition using NC9, FG3019 a human monoclonal antibody targeting connective tissue growth factor (CTGF).

## Data Availability

Not applicable.

## References

[1] Pantalacci S., Tapon N., Léopold P. (2003). The Salvador partner Hippo promotes apoptosis and cell-cycle exit in Drosophila. Nature.

[2] Janse van Rensburg H.J., Azad T., Ling M., Hao Y., Snetsinger B., Khanal P., Minassian L.M., Graham C.H., Rauh M.J., Yang X. (2018). The hippo pathway component taz promotes immune evasion in human cancer through PD-L1. Cancer Res.

[3] Murakami S., Shahbazian D., Surana R., Zhang W., Chen H., Graham G.T., White S.M., Weiner L.M., Yi C. (2017). Yes-associated protein mediates immune reprogramming in pancreatic ductal adenocarcinoma. Oncogene.

[4] Guo X., Zhao Y., Yan H., Yang Y., Shen S., Dai X., Ji X., Ji F., Gong X.G., Li L. (2017). Single tumor-initiating cells evade immune clearance by recruiting type II macrophages. Genes Dev..

[5] Chen P., Zhao D., Li J., Liang X., Li J., Chang A., Henry V.K., Lan Z., Spring D.J., Rao G. (2019). Symbiotic Macrophage-Glioma Cell Interactions Reveal Synthetic Lethality in PTEN-Null Glioma. Cancer Cell.

[6] An Y., Adams J.R., Hollern D.P., Zhao A., Chang S.G., Gams M.S., Chung P.E.D., He X., Jangra R., Shah J.S. (2018). Cdh1 and Pik3ca Mutations Cooperate to Induce Immune-Related Invasive Lobular Carcinoma of the Breast. Cell Rep..

[7] Feng Y., Liang Y., Zhu X., Wang M., Gui Y., Lu Q., Gu M., Xue X., Sun X., He W. (2018). The signaling protein Wnt5a promotes TGFβ1-mediated macrophage polarization and kidney fibrosis by inducing the transcriptional regulators Yap/Taz. J. Biol. Chem..

[8] Fuhrmann G., Herrmann I.K., Stevens M.M. (2015). Cell-derived vesicles for drug therapy and diagnostics: Opportunities and challenges. Nano Today.

[9] Maas S.L.N., Breakefield X.O., Weaver A.M. (2017). Extracellular Vesicles: Unique Intercellular Delivery Vehicles. Trends Cell Biol..

[10] Skog J., Würdinger T., Van Rijn S., Meijer D.H., Gainche L., Sena-Esteves M., Curry W.T., Carter B.S., Krichevsky A.M., Breakefield X.O. (2008). Glioblastoma microvesicles transport RNA and proteins that promote tumour growth and provide diagnostic biomarkers. Nat. Cell Biol..

[11] Al-Nedawi K., Meehan B., Micallef J., Lhotak V., May L., Guha A., Rak J. (2008). Intercellular transfer of the oncogenic receptor EGFRvIII by microvesicles derived from tumour cells. Nat. Cell Biol..

[12] Al-Nedawi K., Meehan B., Kerbel R.S., Allison A.C., Rak J. (2009). Endothelial expression of autocrine VEGF upon the uptake of tumor-derived microvesicles containing oncogenic EGFR. Proc. Natl. Acad. Sci. USA.

[13] Kosaka N., Iguchi H., Hagiwara K., Yoshioka Y., Takeshita F., Ochiya T. (2013). Neutral Sphingomyelinase 2 (nSMase2)-dependent Exosomal Transfer of Angiogenic MicroRNAs Regulate Cancer Cell Metastasis. J. Biol. Chem..

[14] Webber J., Steadman R., Mason M.D., Tabi Z., Clayton A. (2010). Cancer Exosomes Trigger Fibroblast to Myofibroblast Differentiation. Cancer Res..

[15] Wieckowski E.U., Visus C., Szajnik M., Szczepanski M.J., Storkus W.J., Whiteside T.L. (2009). Tumor-Derived Microvesicles Promote Regulatory T Cell Expansion and Induce Apoptosis in Tumor-Reactive Activated CD8+T Lymphocytes. J. Immunol..

[16] Moroishi T., Hayashi T., Pan W.W., Fujita Y., Holt M.V., Qin J., Carson D.A., Guan K.L. (2016). The Hippo Pathway Kinases LATS1/2 Suppress Cancer Immunity. Cell.

[17] Wang G., Lu X., Dey P., Deng P., Wu C.C., Jiang S., Fang Z., Zhao K., Konaparthi R., Hua S. (2015). Targeting YAP-Dependent MDSC Infiltration Impairs Tumor Progression. Cancer Discov..

[18] Sarkar S., Bristow C.A., Dey P., Rai K., Perets R., Ramirez-Cardenas A., Malasi S., Huang-Hobbs E., Haemmerle M., Wu S.Y. (2017). PRKCI promotes immune suppression in ovarian cancer. Genes Dev..

[19] Austenaa L., Natoli G. (2017). A shortcut for early macrophage recruitment into tumors by activated oncogenes. Genes Dev..

[20] Huang Y.-J., Yang C.-K., Wei P.-L., Huynh T.-T., Whang-Peng J., Meng T.-C., Hsiao M., Tzeng Y.-M., Wu A.T., Yen Y. (2017). Ovatodiolide suppresses colon tumorigenesis and prevents polarization of M2 tumor-associated macrophages through YAP oncogenic pathways. J. Hematol. Oncol..

[21] Taha Z., Van Rensburg H.J.J., Yang X. (2018). The Hippo Pathway: Immunity and Cancer. Cancers.

[22] Lee B.S., Park D.I., Lee D.H., Lee J.E., Yeo M.-K., Park Y.H., Lim D.S., Choi W., Lee D.H., Yoo G. (2017). Hippo effector YAP directly regulates the expression of PD-L1 transcripts in EGFR-TKI-resistant lung adenocarcinoma. Biochem. Biophys. Res. Commun..

[23] Tung J.-N., Lin P.-L., Wang Y.-C., Wu D.-W., Chen C.-Y., Lee H. (2018). PD-L1 confers resistance to EGFR mutation-independent tyrosine kinase inhibitors in non-small cell lung cancer via upregulation of YAP1 expression. Oncotarget.

[24] Kim M.H., Kim C.G., Kim S.K., Shin S.J., Choe E.-A., Park S.-H., Shin E.-C., Kim J. (2018). YAP-Induced PD-L1 Expression Drives Immune Evasion in BRAFi-Resistant Melanoma. Cancer Immunol. Res..

[25] Hsu P.C., Miao J., Wang Y.C., Zhang W.Q., Yang Y.L., Wang C.W., Yang C.T., Huang Z., You J., Xu Z. (2018). Inhibition of yes-associated protein down-regulates PD-L1 (CD274) expression in human malignant pleural mesothelioma. J. Cell. Mol. Med..

[26] Taniguchi K., Moroishi T., de Jong P.R., Krawczyk M., Grebbin B.M., Luo H., Xu R.-H., Golob-Schwarzl N., Schweiger C., Wang K. (2017). YAP–IL-6ST autoregulatory loop activated on APC loss controls colonic tumorigenesis. Proc. Natl. Acad. Sci. USA.

[27] Kim W., Khan S.K., Liu Y., Xu R., Park O., He Y., Cha B., Gao B., Yang Y. (2017). Hepatic Hippo signaling inhibits protumoural microenvironment to suppress hepatocellular carcinoma. Gut.

[28] Zhang Q., Zhou R., Xu P. (2020). The Hippo Pathway in Innate Anti-microbial Immunity and Anti-tumor Immunity. Front. Immunol..

[29] Bailey S.R., Nelson M.H., Himes R.A., Zihai L., Mehrotra S., Paulos C.M. (2014). Th17 cells in cancer: The ultimate identity crisis. Front. Immunol..

[30] Geng J., Yu S., Zhao H., Sun X., Li X., Wang P., Xiong X., Hong L., Xie C., Gao J. (2017). The transcriptional coactivator TAZ regulates reciprocal differentiation of TH17 cells and Treg cells. Nat. Immunol..

[31] Lin K.C., Park H.W., Guan K.L. (2018). Deregulation and Therapeutic Potential of the Hippo Pathway in Cancer. Annu. Rev. Cancer Biol..

[32] Yasuda K., Ueda Y., Ozawa M., Matsuda T., Kinashi T. (2016). Enhanced cytotoxic T-cell function and inhibition of tumor progression by Mst1 deficiency. FEBS Lett..

[33] Fan Y., Gao Y., Rao J., Wang K., Zhang F., Zhang C. (2017). YAP-1 Promotes Tregs Differentiation in Hepatocellular Carcinoma by Enhancing TGFBR2 Transcription. Cell. Physiol. Biochem. Int. J. Exp. Cell. Physiol. Biochem. Pharmacol..

[34] Buglioni S., Vici P., Sergi D., Pizzuti L., Di Lauro L., Antoniani B., Sperati F., Terrenato I., Carosi M., Gamucci T. (2016). Analysis of the hippo transducers TAZ and YAP in cervical cancer and its microenvironment. Oncoimmunology.

[35] Ueda Y., Kondo N., Kinashi T. (2020). MST1/2 balance immune activation and tolerance by orchestrating adhesion, transcription, and organelle dynamics in lymphocytes. Front. Immunol..

[36] Rao R.R., Li Q., Gubbels Bupp M.R., Shrikant P.A. (2012). Transcription factor FoxO1 represses T-bet-mediated effector functions and promotes memory CD8+ T cell differentiation. Immunity.

[37] Bouchard A., Witalis M., Chang J., Panneton V., Li J., Bouklouch Y., Suh W.K. (2020). Hippo Signal Transduction Mechanisms in T Cell Immunity. Immune Netw..

[38] Ni X., Tao J., Barbi J., Chen Q., Park B.V., Li Z., Zhang N., Lebid A., Ramaswamy A., Wei P. (2018). YAP Is Essential for Treg-Mediated Suppression of Antitumor Immunity. Cancer Discov..

[39] Tao H., Lu L., Xia Y., Dai F., Wang Y., Bao Y., Lundy S., Ito F., Pan Q., Zhang X. (2014). Antitumor effector B cells directly kill tumor cells via the Fas/FasL pathway and are regulated by IL-10. Eur. J. Immunol..

[40] Kaliss N. (1958). Immunological enhancement of tumor homografts in mice: A review. Cancer Res..

[41] Arima H., Nishikori M., Otsuka Y., Kishimoto W., Izumi K., Yasuda K., Yoshimoto T., Takaori-Kondo A. (2018). B cells with aberrant activation of Notch1 signaling promote Treg and Th2 cell–dominant T-cell responses via IL-33. Blood Adv..

[42] Bai X., Huang L., Niu L., Zhang Y., Wang J., Sun X., Jiang H., Zhang Z., Miller H., Tao W. (2016). Mst1 positively regulates B-cell receptor signaling via CD19 transcriptional levels. Blood Adv..

[43] Lee P.C., Machner M.P. (2018). The legionella effector kinase LegK7 hijacks the host Hippo pathway to promote infection. Cell Host Microbe.

[44] Zhang Y.-L., Li Q., Yang X.-M., Fang F., Li J., Wang Y.-H., Yang Q., Zhu L., Nie H.-Z., Zhang X. (2018). SPON2 Promotes M1-like Macrophage Recruitment and Inhibits Hepatocellular Carcinoma Metastasis by Distinct Integrin–Rho GTPase–Hippo Pathways. Cancer Res..

[45] Zhou Q., Peng R.-Q., Wu X.-J., Xia Q., Hou J.-H., Ding Y., Zhou Q.-M., Zhang X., Pang Z.-Z., Wan D.-S. (2010). The density of macrophages in the invasive front is inversely correlated to liver metastasis in colon cancer. J. Transl. Med..

[46] Edin S., Wikberg M.L., Dahlin A.M., Rutegård J., Öberg Å., Oldenborg P.-A., Palmqvist R. (2012). The Distribution of Macrophages with a M1 or M2 Phenotype in Relation to Prognosis and the Molecular Characteristics of Colorectal Cancer. PLoS ONE.

[47] Zhu P., Baek S.H., Bourk E.M., Ohgi K.A., Garcia-Bassets I., Sanjo H., Akira S., Kotol P.F., Glass C.K., Rosenfeld M.G. (2006). Macrophage/Cancer Cell Interactions Mediate Hormone Resistance by a Nuclear Receptor Derepression Pathway. Cell.

[48] Cui X., Morales R.-T.T., Qian W., Wang H., Gagner J.-P., Dolgalev I., Placantonakis D., Zagzag D., Cimmino L., Snuderl M. (2018). Hacking macrophage-associated immunosuppression for regulating glioblastoma angiogenesis. Biomaterials.

[49] Basu S., Totty N.F., Irwin M.S., Sudol M., Downward J. (2003). Akt Phosphorylates the Yes-Associated Protein, YAP, to Induce Interaction with 14-3-3 and Attenuation of p73-Mediated Apoptosis. Mol. Cell.

[50] Pollard J.W. (2004). Tumour-educated macrophages promote tumour progression and metastasis. Nat. Rev. Cancer.

[51] Mantovani A. (2010). The growing diversity and spectrum of action of myeloid-derived suppressor cells. Eur. J. Immunol..

[52] Talmadge J.E., Gabrilovich D.I. (2013). History of myeloid-derived suppressor cells. Nat. Rev. Cancer.

[53] Yang R., Cai T.-T., Wu X.-J., Liu Y.-N., He J., Zhang X.-S., Ma G., Li J. (2018). Tumour YAP1 and PTEN expression correlates with tumour-associated myeloid suppressor cell expansion and reduced survival in colorectal cancer. Immunology.

[54] OuYang L.-Y., Wu X.-J., Ye S.-B., Zhang R.-X., Li Z.-L., Liao W., Pan Z.-Z., Zheng L.-M., Zhang X.-S., Wang Z. (2015). Tumor-induced myeloid-derived suppressor cells promote tumor progression through oxidative metabolism in human colorectal cancer. J. Transl. Med..

[55] Chun E., Lavoie S., Michaud M., Gallini C.A., Kim J., Soucy G., Odze R., Glickman J.N., Garrett W.S. (2015). CCL2 Promotes Colorectal Carcinogenesis by Enhancing Polymorphonuclear Myeloid-Derived Suppressor Cell Population and Function. Cell Rep..

[56] Wang Y., Justilien V., Brennan K.I., Jamieson L., Murray N.R., Fields A.P. (2017). PKCiota regulates nuclear YAP1 localization and ovarian cancer tumorigenesis. Oncogene.

[57] Kulbe H., Thompson R., Wilson J.L., Robinson S.D., Hagemann T., Fatah R., Gould D., Ayhan A., Balkwill F.R. (2007). The Inflammatory Cytokine Tumor Necrosis Factor-α Generates an Autocrine Tumor-Promoting Network in Epithelial Ovarian Cancer Cells. Cancer Res..

[58] Zhao X., Rong L., Zhao X., Li X., Liu X., Deng J., Wu H., Xu X., Erben U., Wu P. (2012). TNF signaling drives myeloid-derived suppressor cell accumulation. J. Clin. Investig..

[59] Meng G., Wei J., Wang Y., Qu D., Zhang J. (2020). miR-21 regulates immunosuppression mediated by myeloid-derived suppressor cells by impairing RUNX1-YAP interaction in lung cancer. Cancer Cell Int..

[60] Ren W., Hou J., Yang C., Wang H., Wu S., Wu Y., Zhao X., Lu C. (2019). Extracellular vesicles secreted by hypoxia pre-challenged mesenchymal stem cells promote non-small cell lung cancer cell growth and mobility as well as macrophage M2 polarization via miR-21-5p delivery. J. Exp. Clin. Cancer Res..

[61] Maeda R., Sato S., Obata S., Ohno T., Hashiya K., Bando T., Sugiyama H. (2019). Molecular Characteristics of DNA-Alkylating PI Polyamides Targeting RUNX Transcription Factors. J. Am. Chem. Soc..

[62] Kulkarni M., Tan T.Z., Syed Sulaiman N.B., Lamar J.M., Bansal P., Cui J., Qiao Y., Ito Y. (2018). RUNX1 and RUNX3 protect against YAP-mediated EMT, stemness and shorter survival outcomes in breast cancer. Oncotarget..

[63] Pobbati A.V., Hong W. (2020). A combat with the YAP/TAZ-TEAD oncoproteins for cancer therapy. Theranostics.

[64] Liu-Chittenden Y., Huang B., Shim J.S., Chen Q., Lee S.-J., Anders R.A., Liu J.O., Pan D. (2012). Genetic and pharmacological disruption of the TEAD-YAP complex suppresses the oncogenic activity of YAP. Genes Dev..

[65] Holden J., Cunningham C. (2018). Targeting the Hippo Pathway and Cancer through the TEAD Family of Transcription Factors. Cancers.

[66] Barth M.C.S., Montalbetti C., Spitzer L. (2017). Preparation of New 4-(1,1-Dioxo-1,2-benzothiazol-3-yl)hydrazono]methyl]-2-methoxyphenols as Inhibitors of the YAP/TAZ-TEAD Interaction and Their Use in the Treatment of Malignant Mesothelioma. U.S. Patent.

[67] Jiao S., Wang H., Shi Z., Dong A., Zhang W., Song X., He F., Wang Y., Zhang Z., Wang W. (2014). A peptide mimicking VGLL4 function acts as a YAP antagonist therapy against gastric cancer. Cancer Cell.

[68] Pobbati A.V., Chan S.W., Lee I., Song H., Hong W. (2012). Structural and Functional Similarity between the Vgll1-TEAD and the YAP-TEAD Complexes. Structure.

[69] Pobbati A.V., Hong W. (2013). Emerging roles of TEAD transcription factors and its coactivators in cancers. Cancer Biol. Ther..

[70] Aea K. Discovery of a First-in-Class TEAD Inhibitor Which Directly Inhibits YAP/TAZ-TEAD Protein-Protein Interaction and Shows a Potent Anti-Tumor Effect in Malignant Pleural Mesothelioma. Proceedings of the Abstract 3086 AACR Annual Meeting.

[71] Sakamoto K.M., Kim K.B., Kumagai A., Mercurio F., Crews C.M., Deshaies R.J. (2001). Protacs: Chimeric molecules that target proteins to the Skp1-Cullin-F box complex for ubiquitination and degradation. Proc. Natl. Acad. Sci. USA.

[72] Sorrentino G., Ruggeri N., Specchia V., Cordenonsi M., Mano M., Dupont S., Manfrin A., Ingallina E., Sommaggio R., Piazza S. (2014). Metabolic control of YAP and TAZ by the mevalonate pathway. Nat. Cell Biol..

[73] Oku Y., Nishiya N., Shito T., Yamamoto R., Yamamoto Y., Oyama C., Uehara Y. (2015). Small molecules inhibiting the nuclear localization of YAP/TAZ for chemotherapeutics and chemosensitizers against breast cancers. FEBS Open Bio.

[74] Matusewicz L., Meissner J., Toporkiewicz M., Sikorski A.F. (2015). The effect of statins on cancer cells—Review. Tumor Biol..

[75] Gibault F., Sturbaut M., Bailly F., Melnyk P., Cotelle P. (2018). Targeting Transcriptional Enhanced Associate Domains (TEADs). J. Med. Chem..

[76] Zhou T., Zhuang L., Hu Y., Zhou Y., Lin W., Wang D., Wan Z., Chang L., Chen Y., Ying M. (2016). Inactivation of hypoxia-induced YAP by statins overcomes hypoxic resistance tosorafenib in hepatocellular carcinoma cells article. Sci. Rep..

[77] Lee T.F., Tseng Y.C., Nguyen P.A., Li Y.C., Ho C.C., Wu C.W. (2018). Enhanced YAP expression leads to EGFR TKI resistance in lung adenocarcinomas. Sci. Rep..

[78] Liu B.S., Xia H.W., Zhou S., Liu Q., Tang Q.L., Bi N.X., Zhou J.T., Gong Q.Y., Nie Y.Z., Bi F. (2018). Inhibition of YAP reverses primary resistance to EGFR inhibitors in colorectal cancer cells. Oncol. Rep..

[79] Zhao X., Wang X., Fang L., Lan C., Zheng X., Wang Y., Zhang Y., Han X., Liu S., Cheng K. (2017). A combinatorial strategy using YAP and pan-RAF inhibitors for treating KRAS-mutant pancreatic cancer. Cancer Lett..

[80] Lee J.E., Park H.S., Lee D., Yoo G., Kim T., Jeon H., Yeo M.K., Lee C.S., Moon J.Y., Jung S.S. (2016). Hippo pathway effector YAP inhibition restores the sensitivity of EGFR-TKI in lung adenocarcinoma having primary or acquired EGFR-TKI resistance. Biochem. Biophys. Res. Commun..

[81] Lin C.H., Pelissier F.A., Zhang H., Lakins J., Weaver V.M., Park C., LaBarge M.A. (2015). Microenvironment rigidity modulates responses to the HER2 receptor tyrosine kinase inhibitor lapatinib via YAP and TAZ transcription factors. Mol. Biol. Cell.

[82] Mao B., Hu F., Cheng J., Wang P., Xu M., Yuan F., Meng S., Wang Y., Yuan Z., Bi W. (2014). SIRT1 regulates YAP2-mediated cell proliferation and chemoresistance in hepatocellular carcinoma. Oncogene.

[83] Hata S., Hirayama J., Kajiho H., Nakagawa K., Hata Y., Katada T., Furutani-Seiki M., Nishina H. (2012). A novel acetylation cycle of transcription co-activator yes-associated protein that is downstream of hippo pathway is triggered in response to S N2 alkylating agents. J. Biol. Chem..

[84] Galli G.G., Carrara M., Yuan W.C., Valdes-Quezada C., Gurung B., Pepe-Mooney B., Zhang T., Geeven G., Gray N.S., de Laat W. (2015). YAP Drives Growth by Controlling Transcriptional Pause Release from Dynamic Enhancers. Mol. Cell.

[85] Zanconato F., Forcato M., Battilana G., Azzolin L., Quaranta E., Bodega B., Rosato A., Bicciato S., Cordenonsi M., Piccolo S. (2015). Genome-wide association between YAP/TAZ/TEAD and AP-1 at enhancers drives oncogenic growth. Nat. Cell Biol..

[86] Stein C., Bardet A.F., Roma G., Bergling S., Clay I., Ruchti A., Agarinis C., Schmelzle T., Bouwmeester T., Schübeler D. (2015). YAP1 Exerts Its Transcriptional Control via TEAD-Mediated Activation of Enhancers. PLoS Genet..

[87] Levy D., Adamovich Y., Reuven N., Shaul Y. (2008). Yap1 Phosphorylation by c-Abl Is a Critical Step in Selective Activation of Proapoptotic Genes in Response to DNA Damage. Mol. Cell.

[88] Cottini F., Hideshima T., Xu C., Sattler M., Dori M., Agnelli L., ten Hacken E., Bertilaccio M.T., Antonini E., Neri A. (2014). Rescue of Hippo coactivator YAP1 triggers DNA damage-induced apoptosis in hematological cancers. Nat. Med..

